# 1,4-Bis((9H-Carbazol-9-yl)Methyl)Benzene-Containing Electrochromic Polymers as Potential Electrodes for High-Contrast Electrochromic Devices

**DOI:** 10.3390/polym14061175

**Published:** 2022-03-15

**Authors:** Chung-Wen Kuo, Jui-Cheng Chang, Li-Ting Lee, Yi-Dong Lin, Pei-Ying Lee, Tzi-Yi Wu

**Affiliations:** 1 Department of Chemical and Materials Engineering, National Kaohsiung University of Science and Technology, Kaohsiung 80778, Taiwan; welly@nkust.edu.tw (C.-W.K.); a5202350@gmail.com (Y.-D.L.); 2 Department of Chemical Engineering and Materials Engineering, National Yunlin University of Science and Technology, Yunlin 64002, Taiwan; d700215@gmail.com (J.-C.C.); leepeiying1018@gmail.com (P.-Y.L.); 3 Bachelor Program in Interdisciplinary Studies, National Yunlin University of Science and Technology, Yunlin 64002, Taiwan; 4 Department of Materials Science and Engineering, Feng Chia University, Taichung 40724, Taiwan; ltlee@fcu.edu.tw

**Keywords:** electrosynthesis, redox process, color-bleach kinetics, electrochromic device, electrochemical redox stability

## Abstract

Four 1,4-bis((9H-carbazol-9-yl)methyl)benzene-containing polymers (PbCmB, P(bCmB-*co*-bTP), P(bCmB-*co*-dbBT), and P(bCmB-*co*-TF)) were electrosynthesized onto ITO transparent conductive glass and their spectral and electrochromic switching performances were characterized. The PbCmB film displayed four types of color variations (bright gray, dark gray, dark khaki, and dark olive green) from 0.0 to 1.2 V. P(bCmB-*co*-bTP) displayed a high transmittance variation (∆*T* = 39.56% at 685 nm) and a satisfactory coloration efficiency (*η* = 160.5 cm^2^∙C^−1^ at 685 nm). Dual-layer organic electrochromic devices (ECDs) were built using four bCmB-containing polycarbazoles and poly(3,4-ethylenedioxythiophene) (PEDOT) as anodes and a cathode, respectively. PbCmB/PEDOT ECD displayed gainsboro, dark gray, and bright slate gray colors at −0.4 V, 1.0 V, and 2.0 V, respectively. The P(bCmB-*co*-bTP)/PEDOT ECD showed a high ∆*T* (40.7% at 635 nm) and a high coloration efficiency (*η* = 428.4 cm^2^∙C^−1^ at 635 nm). The polycarbazole/PEDOT ECDs exhibited moderate open circuit memories and electrochemical redox stability. The characterized electrochromic properties depicted that the as-prepared polycarbazoles had a satisfactory application prospect as an electrode for the ECDs.

## 1. Introduction

Electrochromism refers to a phenomenon exhibited by certain electroactive species with reversible changes of color and absorption spectra in response to an applied voltage. Many classes of compounds and materials have been shown to exhibit electrochromism over the past four decades. Inorganic transition metal oxides (e.g., WO_3_, TiO_2_, V_2_O_5_, and NiO), inorganic coordination complexes (e.g., Prussian blue), π–conjugated polymers, and molecular dyes are the most frequently studied electrochromic materials [1,2,3,4,5]. π–conjugated polymers have recently attracted an increasing interest due to their superior benefits such as their ease of solution processing, rapid switching speeds, high coloration efficiency, and rich color palette during usage for electrochromic devices. π–conjugated polymers have a wide range of optical and electrochemical applications in field-effect transistors [6,7], capacitors [8,9], photovoltaic cells [10,11], light-emitting diodes [12,13], catalyst supports [14,15], and sensors [16,17].

Nowadays, the most popular π–conjugated polymers (e.g., polythiophenes [18], polyaniline [19,20], polyindoles [21], polycarbazole [22,23], polyfurans [24], polypyrenes [25], and PEDOT-PSS [26]) are widely applied to organic electrochromic devices. Among the many promising π–conjugated polymers, carbazole-containing polymers have attractive optoelectronic properties due to their hole-transporting characteristics via the radical cation and dication species as well as their ease of oxidation through the nitrogen centers. Moreover, polycarbazoles can easily be functionalized at the *N*-, 3,6-, and 2,7-positions, which brings further benefits to modify the electrochromic properties of the resulting polymers. For instance, Ak et al. reported the electrochromic performances of a disulfide-linked polycarbazole (PCS). The PCS was electrosynthesized in BFEE-containing and BFEE-free electrolytic solutions. The PCS prepared in the BFEE-containing electrolytic solution had a high optical contrast (62.5%) and a low response time of 2.0 s [27]. Niu et al. synthesized a series of phenylcarbazole-containing polyimides (PI-6A, PI-6B, PI-6C, PI-6D, and PI-6E). The PI-6A film changed from light yellow (0.0 V), to brown yellow (0.8 V), to deep yellow (1.1 V). The ∆*T* and coloration efficiency of PI-6D were 57% and 250 cm^2^∙C^−1^, respectively, at 568 nm. In addition, five polyimides had a high retained electroactivity (≥88%) after 600 cycles [28]. Poly(3,4-ethylenedioxythiophene) (PEDOT) is a derivative of polythiophenes. PEDOT displays a satisfactory electrochemical reversibility and a high transmittance modulation between its oxidized and neutral states [29]. PEDOT is transparent or dark blue in its oxidized or neutral states, respectively. Accordingly, PEDOT is a potential cathodically coloring layer. Another method is copolymerization (or blending), which is a facile technique for tuning the electrochromic properties of conjugated polymers. The monomers used in copolymerization are bithiophene, 3,3′-dibromo-2,2′-bithiophene, and 2-(thiophen-2-yl)furan. The conjugated length of 2,2′-bithiophene is larger than that of a single thiophene ring; the *E*_onset_ of 2,2′-bithiophene is smaller than that of a single thiophene ring [30]. 3,3′-dibromo-2,2′-bithiophene comprises two additional bromide substituents in the 2,2′-bithiophene unit. Electron-withdrawing bromide substituents increase the *E*_onset_ of the polymers. 2-(thiophen-2-yl)furan contains the optical and electrochemical properties of thiophene and furan; the conjugated polymer chain length of 2-(thiophen-2-yl)furan is greater than those of thiophene and furan.

The use of polymonocarbazole derivatives in electrochromic applications is limited due to their low transmittance change and poor film-forming characteristics. Recently, conjugated polymers with a high transmittance change and good film-forming quality have been achieved by electropolymerizing two or more carbazole-containing monomers [31]. In this present work, a biscarbazole-containing derivative (bCmB) was synthesized and its corresponding homopolymer (PbCmB) and copolymers (P(bCmB-*co*-bTP), P(bCmB-*co*-dbBT), and P(bCmB-*co*-TF)) were electrocoated onto ITO glass substrates. Two carbazole groups of a bCmB unit were bridged by a phenyl and two methylene groups. The incorporation of the methylene groups into the bCmB unit increased the solubility of the resulting polymer. The electrochemical properties, UV–Vis spectra, and switching kinetics of the anodic polymers were characterized. In addition, four dual-layer organic ECDs were built using PbCmB, P(bCmB-*co*-bTP), P(bCmB-*co*-dbBT), and P(bCmB-*co*-TF) as the anodes and PEDOT as the cathode. The UV–Vis spectra, color transitions, switching time, and long-term redox cycling stability of the four ECDs were comprehensively explored.

## 2. Experiment

### 2.1. Materials

**Figure 1:**
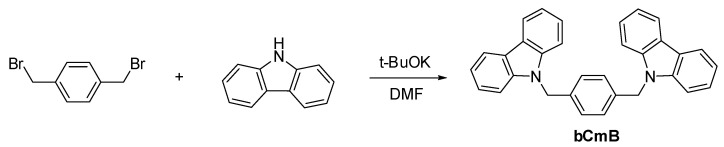
Synthesis of bCmB.

Carbazole, 1,4-bis(bromomethyl)benzene, potassium *tert*-butoxide, and EDOT were purchased from Aldrich, Alfa Aesar, and the Tokyo Chemical Industry, respectively. Dimethyl formamide (DMF) was dried with 4 Å molecular sieves prior to use. The synthetic scheme of bCmB is displayed in Figure 1. The gel electrolyte between the anode and the cathode of the ECDs was prepared using PMMA, LiClO_4_, and PC according to previous procedures [26]. Poly(methyl methacrylate) (PMMA) (*M*_w_ = 350,000; Acros Organics), LiClO_4_, and propylene carbonate (PC) (*M*_w_ = 120; Alfa Aesar) were also commercially available and used as received.

### 2.2. Synthesis of 1,4-Bis((9H-carbazol-9-yl)methyl)benzene (bCmB)

A mixture of carbazole (5.35 g, 32 mmol), 1,4-bis(bromomethyl)benzene (3.96 g, 15 mmol), and 20 mL of DMF was added to a 100 mL two-necked glass reactor. The solution was cooled to 0 °C, treated with potassium *tert*-butoxide (4.26 g, 38 mmol), and then refluxed for 24 h. The quantity of each portion was 7.6 mmol. After cooling, the DMF solvent was evaporated at a reduced pressure (33 mmHg) and an elevated temperature (145 °C) and the remaining mixture was extracted with a 200 mL H_2_O/200 mL CH_2_Cl_2_ mixture. The organic phase (CH_2_Cl_2_ layer) was cleaned with 200 mL brine, further cleaned with 200 mL H_2_O, and then dried using MgSO_4_ for half an hour. The crude product was further purified by column chromatography (silica gels, DCM/n-hexane = 1/2 as an eluent). The mass and yield of the reaction product were 5.04 g and 77%, respectively. The ^1^H NMR (500 MHz, CDCl_3_) results were: *δ* = 5.44 (s, 4H, –NCH_2_); 7.01 (s, 4H, benzene-H); 7.22 (t, J = 7.6 Hz, 4H, carbazole-H); 7.30 (d, J = 7.6 Hz, 4H, carbazole-H); 7.39 (t, J = 7.6 Hz, 4H, carbazole-H); and 8.10 (d, J = 7.6 Hz, 4H, carbazole-H). The ^13^C NMR (175 MHz, DMSO-*d*_6_) results were *δ* = 46.1, 108.8, 119.2, 120.4, 123.0, 125.8, 126.8, 136.5, and 140.5. The elemental analysis calculated (elem. anal. calc.) for C_32_H_24_N_2_ was: C, 88.04%; H, 5.54%; and N, 6.42% with C, 87.92%; H, 5.50%; and N, 6.31% found.

### 2.3. Electrodepositions of PbCmB, PEDOT, P(bCmB-co-bTP), P(bCmB-co-dbBT), and P(bCmB-co-TF) Electrodes

PbCmB, PEDOT, P(bCmB-*co*-bTP), P(bCmB-*co*-dbBT), and P(bCmB-*co*-TF) electrodes were potentiostatically electrodeposited at 1.0 V (vs. Ag/AgNO_3_) on an ITO glass substrate with a charge density of 20 mC∙cm^−2^. The concentration of bCmB, bTP, dbBT, and TF monomers was 2 × 10^−3^ M and the liquid electrolyte was 0.2 M LiClO_4_ in ACN/DCM (1:1 by volume). The feed species and concentrations of the monomers are shown in Table 1. The reference and counter electrodes of the liquid electrolyte cell were an Ag/AgNO_3_ electrode (calibrated with ferrocene/ferrocenium) and a Pt wire, respectively. The polymeric electrode area was 1.5 cm^2^.

### 2.4. Fabrication of the Dual-Layer ECDs

The PMMA/LiClO_4_/PC composite electrolyte was coated onto PbCmB, P(bCmB-*co*-bTP), P(bCmB-*co*-dbBT), and P(bCmB-*co*-TF) anodically coloring films. The cathodically coloring film (PEDOT) was placed onto the PMMA/LiClO_4_/PC layer. The sandwich configuration of the ECDs was anodically coloring film/electrolyte/cathodically coloring film. The active areas of PbCmB, P(bCmB-*co*-bTP), P(bCmB-*co*-dbBT), P(bCmB-*co*-TF), and PEDOT were ca. 1.0 × 1.5 cm^2^.

### 2.5. Electrochemical, Electrochromic, and Kinetic Characterizations

The electrochemical polymerization procedures and electrochemical properties of the PbCmB, P(bCmB-*co*-bTP), P(bCmB-*co*-dbBT), and P(bCmB-*co*-TF) anodically coloring films were carried out using a CHI6277E (CH Instruments, Austin, TX, USA) electrochemical workstation with a scan rate of 100 mV s^−1^. The absorption spectra and electrochromic switching performances of the single-layer electrodes in the solutions and the dual-layer ECDs were measured using a spectrophotometer (Jasco V-630 (JASCO International Co., Ltd., Tokyo, Japan)) and an electrochemical workstation (CHI6277E). The color-bleach kinetics of the polymers were switched between 0.0 and 1.2 V whereas the color-bleach kinetics of the ECDs were switched between −0.4 V and 1.8 V.

## 3. Results and Discussion

### 3.1. Electrochemical Characterization of the Polymer Electrodes

**Figure 2:**
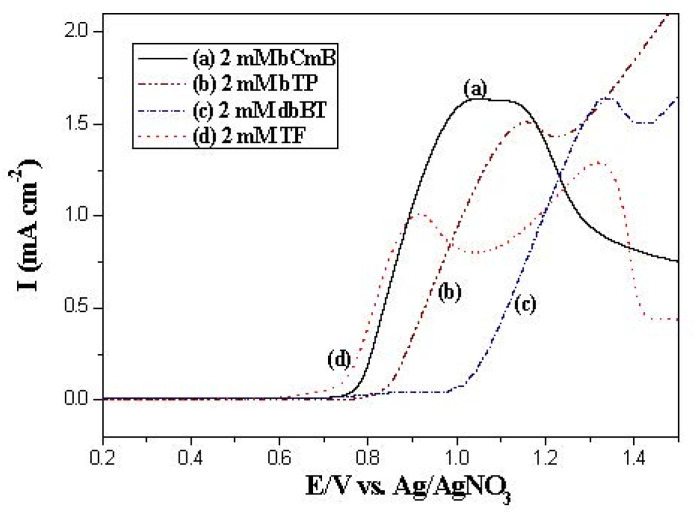
Cyclic voltammograms of (**a**) 2 mM bCmB, (**b**) 2 mM bTP, (**c**) 2 mM dbBT, and (**d**) 2 mM TF containing 0.2 M LiClO_4_ at a scan rate of 100 mV s^−1^.

Figure 2 shows the linear sweep voltammetry curves for the electrochemical oxidation of neat PbCmB, P(bCmB-*co*-bTP), P(bCmB-*co*-dbBT), and P(bCmB-*co*-TF) in a 0.2 M LiClO_4_ solution. The onset potentials in the cyclic voltammetry of neat bCmB, bTP, dbBT, and TF were 0.75, 0.81, 0.94, and 0.70 V (vs. Ag/AgNO_3_), respectively. The incorporation of two electron-withdrawing bromide groups in the bithiophene unit increased the onset potentials. bCmB displayed a lower onset potential than the bithiophene unit; this could be attributed to the fact that biscarbazole-containing bCmB showed a stronger electron-donating ability than the bithiophene unit. The disparities of the onset potentials between neat bCmB and neat bithiophene derivatives were less than 0.2 V, implying the feasibility of copolymerizations using bCmB and bithiophene derivatives.

**Figure 3:**
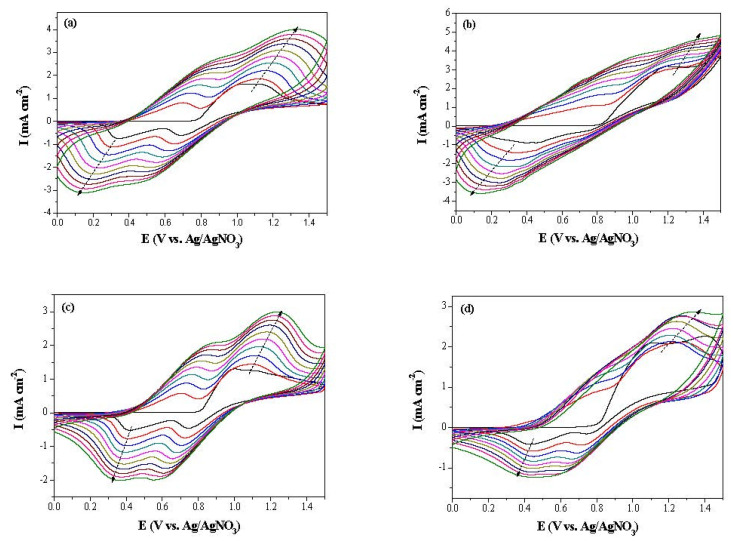
Electrochemical synthesis of (**a**) PbCmB, (**b**) P(bCmB-*co*-bTP), (**c**) P(bCmB-*co*-dbBT), and (**d**) P(bCmB-*co*-TF) in an ACN/DCM (1:1 by volume) solution at 100 mV s^−1^ on ITO glass.

Figure 3 displays the electrogrowth of neat bCmB and mixtures of bCmB + bithiophene derivatives (or 2-(thiophen-2-yl)furan) in 0.2 M LiClO_4_/ACN/DCM). The potentiodynamic polymerization scans from the first to the tenth cycles revealed that the current densities of the redox peaks increased with the number of increasing cycles, implying the growth of polymers on the ITO substrate [32]. As shown in Figure 3a, PbCmB displayed two distinct oxidation peaks at 0.70 and 1.12 V as well as two evident reduction peaks at 0.31 and 0.65 V.

**Figure 4:**
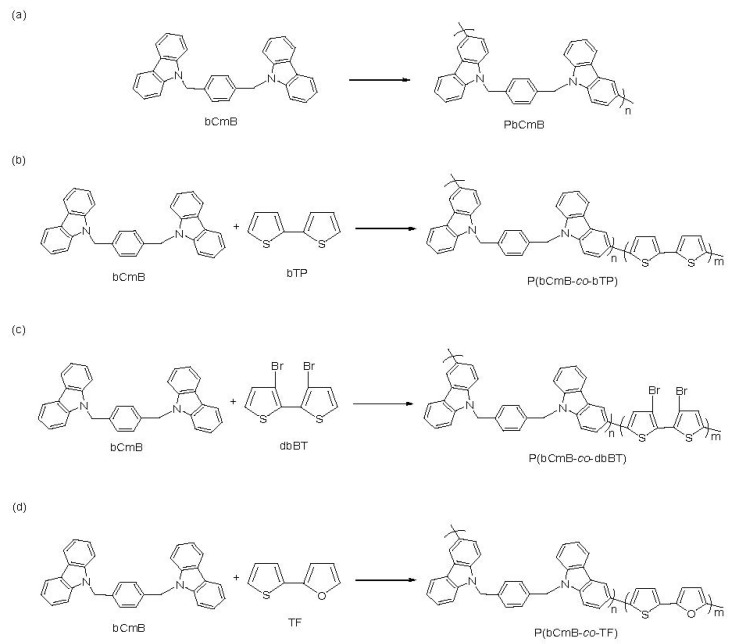
The electrochemical polymerization scheme of (**a**) PbCmB, (**b**) P(bCmB-*co*-bTP), (**c**) P(bCmB-*co*-dbBT), and (**d**) P(bCmB-*co*-TF).

The first oxidation and reduction peaks depicted the generation of radical cations in poly(1,4-bis((9H-carbazol-9-yl)methyl)benzene) and the second redox peaks represented the formation of dications. The incorporation of bithiophene, 3,3′-dibromo-2,2′-bithiophene, and 2-(thiophen-2-yl)furan into the polymeric chain slightly shifted the redox peaks. The first oxidation and reduction peaks of P(bCmB-*co*-bTP) were located at 0.78 and 0.33 V, respectively (Figure 3b). In a similar condition, two oxidation peaks of P(bCmB-*co*-dbBT) were situated at 0.70 and 1.09 V, respectively, and two reduction peaks of P(bCmB-*co*-dbBT) were located at 0.41 and 0.72 V, respectively (Figure 3c). The first and second oxidation peaks of P(bCmB-*co*-TF) were located at 0.79 and 1.26 V, respectively, and the first and second reduction peaks of P(bCmB-*co*-TF) were situated at 0.44 and 0.71 V, respectively (Figure 3d). The peak potentials and CV wave shapes of PbCmB were diverse compared with those of P(bCmB-*co*-bTP), P(bCmB-*co*-dbBT), and P(bCmB-*co*-TF), proving the coating of P(bCmB-*co*-bTP), P(bCmB-*co*-dbBT), and P(bCmB-*co*-TF) membranes onto the ITO glass substrate. The polymerization schemes of PbCmB, P(bCmB-*co*-bTP), P(bCmB-*co*-dbBT), and P(bCmB-*co*-TF) are listed in Figure 4 [33].

**Figure 5:**
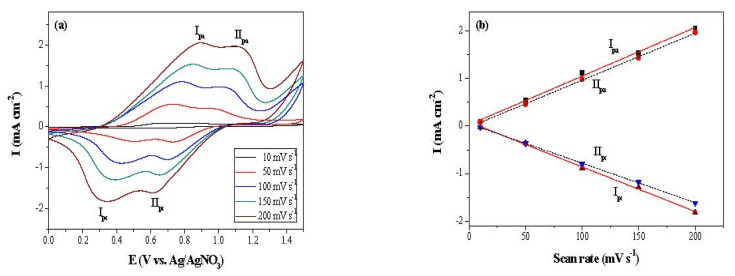
(**a**) CV curves of the P(bCmB-*co*-bTP) film at scan rates between 10 and 200 mV s^−1^ in ACN/DCM (1:1 by volume) containing 0.2 M LiClO_4_. (**b**) Scan rate dependence of anodic and cathodic peak current densities for the P(bCmB-*co*-bTP) film.

The electrocoated P(bCmB-*co*-bTP) film was studied using various scan rate cyclic voltammetry measurements. As displayed in Figure 5, the P(bCmB-*co*-bTP) film showed two couples of well-defined redox peaks at various scan rates in 0.2 M LiClO_4_/ACN/DCM; the peak current densities of the P(bCmB-*co*-bTP) film were linearly proportional to the scan velocities, representing that P(bCmB-*co*-bTP) tightly adhered to the conductive glass and the redox behaviors of P(bCmB-*co*-bTP) were reversible and activation control [34].

### 3.2. Absorption Spectra and the Transmittance Changes of the Polymers

**Figure 6:**
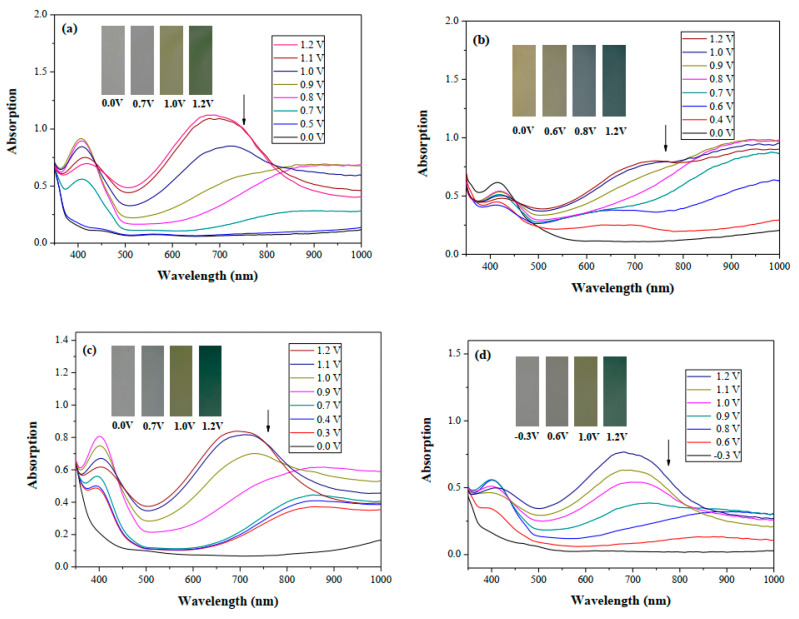
UV–Vis spectra of (**a**) PbCmB, (**b**) P(bCmB-*co*-bTP), (**c**) P(bCmB-*co*-dbBT), and (**d**) P(bCmB-*co*-TF) in an ACN/DCM (1:1 by volume) solution containing 0.2 M LiClO_4_.

Figure 6a–d shows the absorption spectra of the PbCmB, P(bCmB-*co*-bTP), P(bCmB-*co*-dbBT), and P(bCmB-*co*-TF) electrodes in 0.2 M LiClO_4_/ACN/DCM. There was no noticeable absorption peak of the PbCmB film between 370 and 1000 nm at 0.0 and 0.5 V, respectively. As the voltage increased stepwise from 0.0 V to 1.2 V, new charge carrier bands appeared at around 420 and 675 nm, signifying the existence of the generation of radical cations and dications [35] (Figure 6a). As displayed in Figure 6b, P(bCmB-*co*-bTP) showed a neutral absorption peak at around 420 nm; this could be attributed to the π–π* (or n–π*) transition of the bithiophene heteroaromatic groups. The PbCmB film displayed four types of color variations from the neutral to the oxidation state, which were bright gray, dark gray, dark khaki, and dark olive green at 0.0, 0.7, 1.0, and 1.2 V, respectively. The L*, a*, and b* values of PbCmB are displayed in Table 2. Under identical situations, the P(bCmB-*co*-bTP) film was celadon, earth gray, iron gray, and navy blue at 0.0, 0.6, 0.8, and 1.2 V, respectively.

The P(bCmB-*co*-dbBT) film was bright gray, slate gray, khaki, and dark greenish grey at 0.0, 0.7, 1.0, and 1.2 V, respectively. The P(bCmB-*co*-TF) film was light gray, slate gray, dark khaki, and dark greenish grey at −0.3, 0.6, 1.0, and 1.2 V, respectively. The incorporation of bithiophene, 3,3′-dibromo-2,2′-bithiophene, and 2-(thiophen-2-yl)furan in the polymer chain changed the color variations from the reduced state to the oxidized state.

The optical energy gap (*E*_g_) of PbCmB calculated using the absorption onset wavelength *(λ*_onset_) of the π–π* transition peak was 3.20 eV [36]. Table 3 shows the *E*_g_ of the reported polymers. PbCmB displayed a larger *E*_g_ than PBCPO [37], PMCP [38], and PDCP [39]. This could be attributed to two methylene groups interrupting the conjugated degree of the polymer chains. The *E*_onset_ of PbCmB (vs. Ag/AgNO_3_) was 0.80 V, the *E*_FOC_ calculated from the potential of ferrocene/ferrocenium vs. Ag/AgNO_3_ was 0.69 V, and the *E*_onset_ (vs. *E*_FOC_) was evaluated as 0.11 V. The reference energy for ferrocene is 4.8 eV below the vacuum level [40]. *E*_HOMO_ and *E*_LUMO_, corresponding with the energy levels of HOMO and LUMO, were calculated as −4.91 and −1.71 eV, respectively.

**Figure 7:**
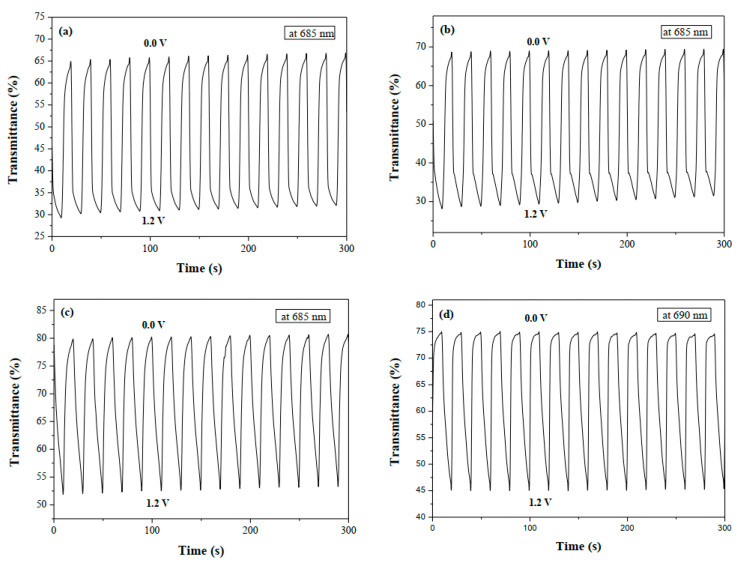
Optical contrast of (**a**) PbCmB, (**b**) P(bCmB-*co*-bTP), (**c**) P(bCmB-*co*-dbBT), and (**d**) P(bCmB-*co*-TF) in an ACN/DCM (1:1 by volume) solution containing 0.2 M LiClO_4_ with a residence time of 10 s.

Figure 7 displays the electrochromic switching profiles of PbCmB, P(bCmB-*co*-bTP), P(bCmB-*co*-dbBT), and P(bCmB-*co*-TF) in the solutions, which changed between 0.0 and 1.2 V with a time interval of 10 s. The transmittance changes (Δ*T*) of PbCmB, P(bCmB-*co*-bTP), P(bCmB-*co*-dbBT), and P(bCmB-*co*-TF) from the neutral state to the oxidized state were determined to be 23.94% at 685 nm, 39.56% at 685 nm, 27.85% at 685 nm, and 29.84% at 690 nm, respectively. P(bCmB-*co*-bTP) revealed the highest Δ*T* among the four electrodes. The copolymers (P(bCmB-*co*-bTP), P(bCmB-*co*-dbBT), and P(bCmB-*co*-TF)) showed a higher Δ*T* than the homopolymer (PbCmB) in the solutions, suggesting that the electrochemical copolymerization of PbCmB with bithiophene, 3,3′-dibromo-2,2′-bithiophene, or 2-(thiophen-2-yl)furan monomer led to an increase in the Δ*T*. P(bCmB-*co*-bTP) displayed a higher Δ*T* than PBCPO [37], PMCP [38], PDCP [39], and PDTCZ-2 [41]. However, the Δ*T* of P(bCmB-*co*-bTP) was smaller than P(DTC-*co*-BTP2) [42].

The response time from the colored to the bleached state (*τ*_b_) and from the bleached to the colored state (*τ*_c_) of PbCmB, P(bCmB-*co*-bTP), P(bCmB-*co*-dbBT), and P(bCmB-*co*-TF) in the solutions is listed in Table 4. The *τ*_b_ and *τ*_c_ were determined at 90% of the maximum Δ*T*. The *τ*_c_ and *τ*_b_ of the polymeric films were determined to be 1.46–3.51 s and 4.81–5.18 s, respectively.

As shown in the following equation [48], the ∆OD could be determined by a logarithmic calculation of the transmission (%) at the oxidation state and the reduction state:

(1) $$\Delta \mathrm{OD}=\log(\frac{T_{\mathrm{ox}}}{T_{\mathrm{re}d}})$$

The ΔODs of the PbCmB, P(bCmB-*co*-bTP), P(bCmB-*co*-dbBT), and P(bCmB-*co*-TF) films in the solutions were 0.126 at 685 nm, 0.374 at 685 nm, 0.186 at 685 nm, and 0.221 at 690 nm, respectively. The P(bCmB-*co*-bTP), P(bCmB-*co*-dbBT), and P(bCmB-*co*-TF) films displayed a higher ΔOD than the PbCmB film.

The coloration efficiency (*η*) could be obtained from the following equation [48]:

(2) $$\eta =\frac{\Delta \mathrm{OD}}{Q_{d}}$$

where *Q*_d_ represents the charge injection/extraction of the electrodes per active area. As listed in Table 4, the *η* of PbCmB, P(bCmB-*co*-bTP), P(bCmB-*co*-dbBT), and P(bCmB-*co*-TF) was 80.3 cm^2^∙C^−1^ at 685 nm, 160.5 cm^2^∙C^−1^ at 685 nm, 86.5 cm^2^∙C^−1^ at 685 nm, and 125.8 cm^2^∙C^−^^1^ at 690 nm, respectively.

### 3.3. Absorption Spectra and the Transmittance Changes of the ECDs

**Figure 8:**
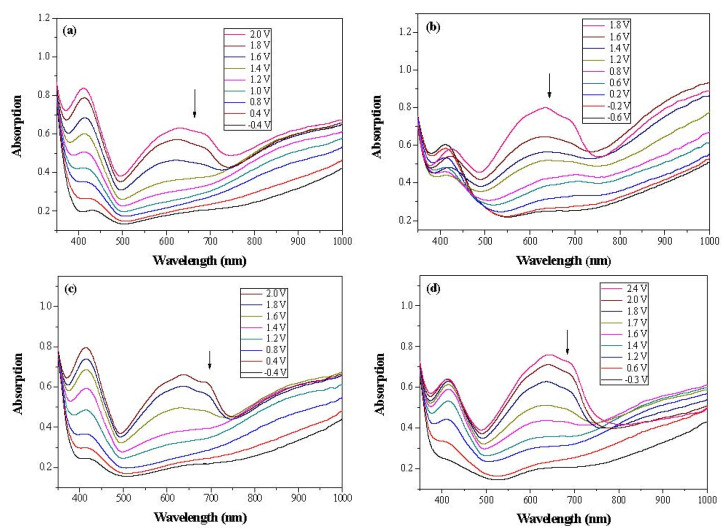
UV–Vis spectra of (**a**) PbCmB/PEDOT, (**b**) P(bCmB-*co*-bTP)/PEDOT, (**c**) P(bCmB-*co*-dbBT)/PEDOT, and (**d**) P(bCmB-*co*-TF)/PEDOT ECDs.

Dual-layer organic ECDs were constructed using the configurations of the PbCmB/PEDOT, P(bCmB-*co*-bTP)/PEDOT, P(bCmB-*co*-dbBT)/PEDOT, and P(bCmB-*co*-TF)/PEDOT ECDs. The absorption spectra of the PbCmB/PEDOT, P(bCmB-*co*-bTP)/PEDOT, P(bCmB-*co*-dbBT)/PEDOT, and P(bCmB-*co*-TF)/PEDOT ECDs are displayed in Figure 8a–d. At −0.4 V, the PbCmB/PEDOT ECD did not reveal a significant absorption peak in the UV-Vis region. The anodic PbCmB film was in its reduced state and the cathodic PEDOT film was in its oxidized state at −0.4 V, exhibiting a limpid color. The PbCmB/PEDOT ECD showed a gainsboro color at −0.4 V. After progressively raising the potential to 2.0 V, the PbCmB film started to oxidize and the PEDOT film started to reduce. As a consequence, new absorption bands at 420 and 620 nm appeared stepwise. The PbCmB/PEDOT ECD showed a dark gray color at 1.0 V and a bright slate gray color at 2.0 V. Under identical situations, the P(bCmB-*co*-bTP)/PEDOT ECD was light gray, dark gray, and slate gray at −0.6, 0.8, and 2.0 V, respectively. The P(bCmB-*co*-dbBT)/PEDOT ECD was gainsboro, dark gray, and bright slate gray at −0.4, 1.0, and 2.4 V, respectively. The P(bCmB-*co*-TF)/PEDOT ECD was gainsboro, light gray, and dark gray at −0.3, 1.6, and 2.4 V, respectively. The colorimetric results of the four ECDs are listed in Table 5.

**Figure 9:**
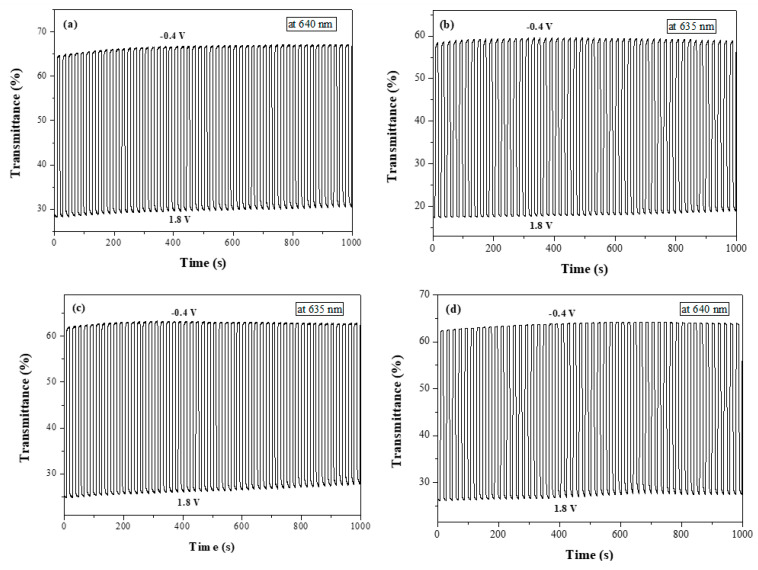
Optical contrast of (**a**) PbCmB/PEDOT, (**b**) P(bCmB-*co*-bTP)/PEDOT, (**c**) P(bCmB-*co*-dbBT)/PEDOT, and (**d**) P(bCmB-*co*-TF)/PEDOT ECDs with a residence time of 10 s.

Figure 9 shows the color-bleach kinetics of the PbCmB/PEDOT, P(bCmB-*co*-bTP)/PEDOT, P(bCmB-*co*-dbBT)/PEDOT, and P(bCmB-*co*-TF)/PEDOT ECDs; the time interval of the cycle switched between −0.4 V and 1.8 V was 10 s. Table 6 lists the Δ*T*, ΔOD, *η*, *τ*_b_, and *τ*_c_ of the four ECDs. The Δ*T* of the PbCmB/PEDOT, P(bCmB-*co*-bTP)/PEDOT, P(bCmB-*co*-dbBT)/PEDOT, and P(bCmB-*co*-TF)/PEDOT ECDs was 35.3% at 640 nm, 40.7% at 635 nm, 36.4% at 635 nm, and 35.7% at 640 nm at the second cycle, respectively. The P(bCmB-*co*-bTP)/PEDOT ECD showed the highest Δ*T*. The P(bCmB-*co*-bTP)/PEDOT, P(bCmB-*co*-dbBT)/PEDOT, and P(bCmB-*co*-TF)/PEDOT ECDs displayed a higher Δ*T* than the PbCmB/PEDOT ECD, indicating that the use of bithiophene, 3,3′-dibromo-2,2′-bithiophene, and 2-(thiophen-2-yl)furan-containing copolymers as the anodically coloring layers in the ECDs gave rise to a higher Δ*T* than the PbCmB. The P(bCmB-*co*-bTP)/PEDOT ECD displayed a higher Δ*T* than the PtCz/PProDOT-Me_2_ [43], P(Bmco)/PEDOT [44], P(dcbp-*co*-cpdt)/PEDOT [45], P(DiCP-*co*-CDTK)/PEDOT-PSS [39], and P(TTPA-*co*-EDOT)/PEDOT ECDs [46]. However, the P(bCmB-*co*-bTP)/PEDOT ECD showed a lower Δ*T* than the P(BCz-*co*-In)/PProDOT-Et_2_ ECD [47].

The *η* of the dual-layer organic ECDs is also shown in Table 6. The *η* of the PbCmB/PEDOT, P(bCmB-*co*-bTP)/PEDOT, P(bCmB-*co*-dbBT)/PEDOT, and P(bCmB-*co*-TF)/PEDOT ECDs was 305.8 cm^2^∙C^−1^ at 640 nm, 428.4 cm^2^∙C^−1^ at 635 nm, 341.0 cm^2^∙C^−1^ at 635 nm, and 316.8 cm^2^∙C^−1^ at 640 nm at the second cycle, respectively. The P(bCmB-*co*-bTP)/PEDOT ECD displayed the highest *η*. The use of P(bCmB-*co*-bTP), P(bCmB-*co*-dbBT), and P(bCmB-*co*-TF) as the anodically coloring materials in the ECDs led to a higher *η* than the PbCmB. Table 3 also summarizes the *η* of the reported ECDs. The P(bCmB-*co*-bTP)/PEDOT ECD showed a higher *η* than the PtCz/PProDOT-Me_2_ [43], P(dcbp-*co*-cpdt)/PEDOT [45], and P(TTPA-*co*-EDOT)/PEDOT ECDs [46]. However, the P(bCmB-*co*-bTP)/PEDOT ECD displayed a lower *η* than the P(BCz-*co*-In)/PProDOT-Et_2_ [47] and P(DiCP-*co*-CDTK)/PEDOT-PSS ECDs [39].

The response time of the PbCmB/PEDOT, P(bCmB-*co*-bTP)/PEDOT, P(bCmB-*co*-dbBT)/PEDOT, and P(bCmB-*co*-TF)/PEDOT ECDs was shorter than the PbCmB, P(bCmB-*co*-bTP), P(bCmB-*co*-dbBT), and P(bCmB-*co*-TF) electrodes in the solutions, disclosing that the distances between the anode and the cathode in the ECDs were narrower than between the polymeric electrode and the Pt electrode in the solutions [49].

### 3.4. Open Circuit Memories of the ECDs

**Figure 10:**
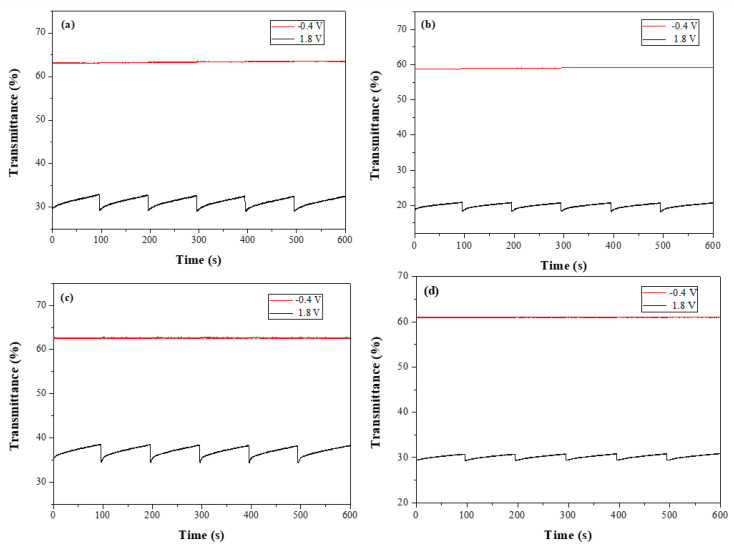
Open circuit stability of the (**a**) PbCmB/PEDOT, (**b**) P(bCmB-*co*-bTP)/PEDOT, (**c**) P(bCmB-*co*-dbBT)/PEDOT, and (**d**) P(bCmB-*co*-TF)/PEDOT ECDs.

The open circuit memories of the dual-layer organic ECDs were monitored by applying potentials in colored and bleached states for 1 s for each 100 s interval. As displayed in Figure 10, the PbCmB/PEDOT, P(bCmB-*co*-bTP)/PEDOT, P(bCmB-*co*-dbBT)/PEDOT, and P(bCmB-*co*-TF)/PEDOT ECDs displayed sufficient open circuit memories with ≤1.1% transmittance variation in the bleached state. However, in an oxidized state of the PbCmB, P(bCmB-*co*-bTP), P(bCmB-*co*-dbBT), and P(bCmB-*co*-TF) films and in a reduced state of the PEDOT film, the transmittance changes of the PbCmB/PEDOT, P(bCmB-*co*-bTP)/PEDOT, P(bCmB-*co*-dbBT)/PEDOT, and P(bCmB-*co*-TF)/PEDOT ECDs in a colored state were less stable than the four ECDs in a bleached state. The P(bCmB-*co*-dbBT)/PEDOT ECD showed the largest transmittance change in a colored state. However, the transmittance change of the P(bCmB-*co*-dbBT)/PEDOT ECD in a colored state was less than 4.9%, implying that the PbCmB/PEDOT, P(bCmB-*co*-bTP)/PEDOT, P(bCmB-*co*-dbBT)/PEDOT, and P(bCmB-*co*-TF)/PEDOT ECDs had ample open circuit memories in both the colored and bleached states.

### 3.5. Long-Term Electrochemical Redox Stability of the ECDs

**Figure 11:**
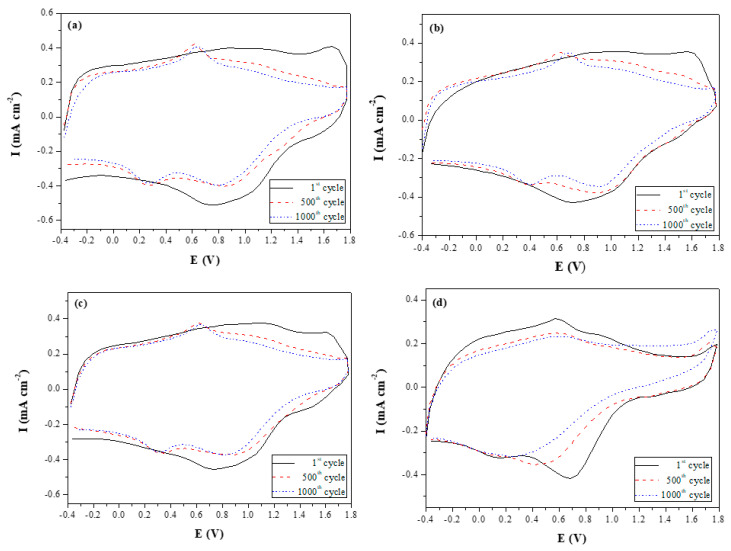
Cyclic voltammograms of (**a**) PbCmB/PEDOT, (**b**) P(bCmB-*co*-bTP)/PEDOT, (**c**) P(bCmB-*co*-dbBT)/PEDOT, and (**d**) P(bCmB-*co*-TF)/PEDOT ECDs as a function of repeated cycles with a scan rate of 500 mV s^−1^ between 1 and 1000 cycles.

The long-term cyclic voltammetry stability of the PbCmB/PEDOT, P(bCmB-*co*-bTP)/PEDOT, P(bCmB-*co*-dbBT)/PEDOT, and P(bCmB-*co*-TF)/PEDOT ECDs was detected for 1000th cycles with a scan speed of 500 mV∙s^−^^1^. As displayed in Figure 11, 84.9%, 92.8%, 85.4%, and 85.7% of the electrochemical activity was maintained at the 500th cycle, respectively, and 75.5%, 81.5%, 78.2%, and 76.6% of the electrochemical activity was maintained at the 1000th cycle for the PbCmB/PEDOT, P(bCmB-*co*-bTP)/PEDOT, P(bCmB-*co*-dbBT)/PEDOT, and P(bCmB-*co*-TF)/PEDOT ECDs, respectively. The P(bCmB-*co*-bTP)/PEDOT, P(bCmB-*co*-dbBT)/PEDOT, and P(bCmB-*co*-TF)/PEDOT ECDs that used copolymers as anodic layers showed a better long-term stability than the PbCmB/PEDOT ECD at the 500th and 1000th cycles.

## 4. Conclusions

A series of redox-active polycarbazoles (PbCmB, P(bCmB-*co*-bTP), P(bCmB-*co*-dbBT), and P(bCmB-*co*-TF)) were electrochemically synthesized onto ITO glass surfaces. The obtained polycarbazoles showed a good redox reversibility. Compared with the homopolymer, the copolymers showed different color transitions from the neutral to the oxidized states. The P(bCmB-*co*-bTP) film was celadon, earth gray, iron gray, and navy blue at 0.0, 0.6, 0.8, and 1.2 V, respectively. Electrochromic switching studies of the four polycarbazoles exhibited that the Δ*T* of PbCmB and P(bCmB-*co*-TF) was 23.94% at 685 nm and 29.84% at 690 nm, respectively. Four ECDs were built using anodic polycarbazoles and a cathodic PEDOT layer. The Δ*T* of the PbCmB/PEDOT and P(bCmB-*co*-dbBT)/PEDOT ECDs was 35.3% at 640 nm and 36.4% at 635 nm at the second cycle, respectively. bCmB-containing polycarbazoles and their corresponding ECDs showed high transmittance changes. The incorporation of bithiophene, 3,3′-dibromo-2,2′-bithiophene, and 2-(thiophen-2-yl)furan groups into the polycarbazoles showed different color transitions at various potentials and showed high transmittance changes. Moreover, five ECDs displayed a fast response time (≤2.8 s) and satisfactory open circuit memories in both the colored and bleached states. As a result, PbCmB and P(bCmB-*co*-bTP) may be good candidates for use as electrodes in ECDs.

## Figures and Tables

**Table 1:** Feed species of polymeric electrodes.

Electrodes	Anodic Polymers	Feed Species of Anodic Polymer	Feed Molar Ratio of Anodic Polymer
(a)	PbCmB	2 mM bCmB	Neat bCmB
(b)	P(bCmB-*co*-bTP)	2 mM bCmB + 2 mM bTP	2:2
(c)	P(bCmB-*co*-dbBT)	2 mM bCmB + 2 mM dbBT	2:2
(d)	P(bCmB-*co*-TF)	2 mM bCmB + 2 mM TF	2:2

**Table 2:** Colorimetric values (L*, a*, and b*), CIE chromaticity values (*x*, *y*), and diagrams of (**a**) PbCmB, (**b**) P(bCmB-*co*-bTP), (**c**) P(bCmB-*co*-dbBT), and (**d**) P(bCmB-*co*-TF) at different potentials.

Potential (V)	L*	a*	b*	*x*	*y*	Diagram
(a)						
0.0	91.76	−7.56	9.74	0.319	0.353	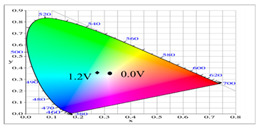
0.7	87.42	−16.01	32.11	0.347	0.407	
0.9	75.15	−25.42	19.31	0.309	0.398	
1.0	64.88	−28.71	7.611	0.277	0.377	
1.2	61.98	−25.31	2.478	0.271	0.359	
(b)						
0.0	87.33	−5.54	32.78	0.365	0.399	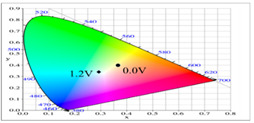
0.4	81.32	−8.09	13.47	0.326	0.364	
0.7	74.83	−11.79	9.61	0.313	0.361	
0.9	70.63	−13.91	6.46	0.303	0.357	
1.2	67.53	−12.56	0.104	0.291	0.340	
(c)						
0.0	92.86	−0.44	5.13	0.321	0.339	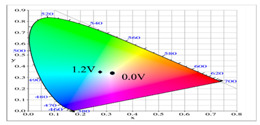
0.5	90.67	−6.87	12.66	0.326	0.359	
0.9	80.32	−16.51	20.19	0.326	0.388	
1.0	72.65	−21.29	10.64	0.299	0.373	
1.2	64.68	−21.34	0.542	0.274	0.349	
(d)						
0.0	97.08	−2.03	8.37	0.324	0.345	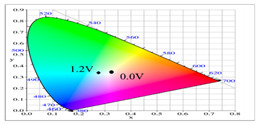
0.6	93.67	−4.99	14.82	0.332	0.361	
0.9	82.78	−14.03	14.19	0.318	0.371	
1.1	71.73	−16.35	0.11	0.285	0.342	
1.2	66.68	−18.55	−2.18	0.274	0.339	

**Table 3:** Transmittance changes and coloration efficiencies of polymers (or ECDs).

Polymers or ECDs	Δ*T*_max_ (%)	*η*_max_ (cm^2^∙C^−1^)	*E*_g_^a^ (eV)	Ref.
PBCPO	30.1 (735nm)	85 (735 nm)	3.14	[37]
PMCP	29 (460 nm)	-	3.14	[38]
PDCP	19 (1025 nm)	124 (1025 nm)	2.58	[39]
PDTCZ-2	30.7 (898 nm)	169 (898 nm)	-	[41]
P(DTC-*co*-BTP2)	68.4 (855 nm)	159.4 (855 nm)	-	[42]
PbCmB	23.94 (685 nm)	80.3 (685 nm)	3.20	This work
P(bCmB-*co*-bTP)	39.56 (685 nm)	160.5 (685 nm)	-	This work
PtCz/PProDOT-Me_2_	36 (572 nm)	343.4 (572 nm)	-	[43]
P(Bmco)/PEDOT	35 (620 nm)	-	-	[44]
P(dcbp-*co*-cpdt)/PEDOT	39.8 (628 nm)	319.98 (628 nm)	-	[45]
P(TTPA-*co*-EDOT)/PEDOT	20 (775 nm)	336 (775 nm)	-	[46]
P(BCz-*co*-In)/PProDOT-Et_2_	42.0 (587 nm)	634 (587 nm)	-	[47]
P(DiCP-*co*-CDTK)/PEDOT-PSS	38 (635 nm)	634 (635 nm)	-	[39]
P(bCmB-*co*-bTP)/PEDOT	40.7 (635 nm)	428.4 (635 nm)	-	This work

**Table 4:** Electrochromic switching properties investigated at specific wavelengths for the electrodes.

Electrodes	λ (nm) ^a^	*T*_ox_	*T*_red_	∆*T*	△OD	Q_d_ (mC cm^−2^)	*η* (cm^2^∙C^−1^)	τ_c_ (s)	τ_b_ (s)
PbCmB	685	71.22	95.16	23.94	0.126	2.78	80.3	1.83	5.10
P(bCmB-*co*-bTP)	685	28.97	68.53	39.56	0.374	4.19	160.5	3.51	4.81
P(bCmB-*co*-dbBT)	685	52.07	79.92	27.85	0.186	3.88	86.5	2.64	5.05
P(bCmB-*co*-TF)	690	45.05	74.89	29.84	0.221	3.25	125.8	1.46	5.18

**^a^** The selected applied wavelength for the electrodes.

**Table 5:** Electrochromic photographs, colorimetric values (L*, a*, and b*), CIE chromaticity values (*x*, *y*), and diagrams of (**a**) PbCmB/PEDOT, (**b**) P(bCmB-*co*-bTP)/PEDOT, (**c**) P(bCmB-*co*-dbBT)/PEDOT, and (**d**) P(bCmB-*co*-TF)/PEDOT ECDs at different potentials.

ECD	Potential (V)	Photographs	L*	a*	b*	*x*	*y*	Diagram
(a)								
PbCmB/PEDOT	−0.4	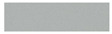	86.69	−5.91	2.73	0.309	0.339	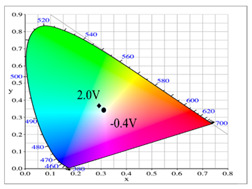
	0.8	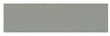	83.53	−9.09	9.36	0.317	0.356	
	1.0	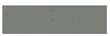	81.24	−10.17	10.88	0.318	0.361	
	1.4	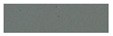	74.73	−13.07	12.32	0.317	0.369	
	2.0	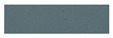	62.04	−18.35	6.08	0.292	0.364	
(b)								
P(bCmB-*co*-bTP)/PEDOT	−0.6	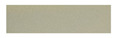	80.63	−9.79	22.19	0.341	0.386	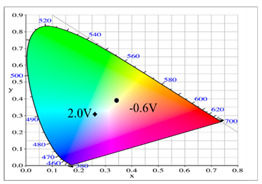
	0.2	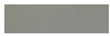	78.28	−10.98	14.81	0.325	0.371	
	0.8	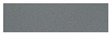	72.73	−9.73	3.32	0.303	0.345	
	1.2	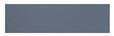	67.11	−8.04	−4.71	0.288	0.324	
	2.0	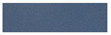	54.98	−10.98	−11.85	0.259	0.304	
(c)								
P(bCmB-*co*-dbBT)/PEDOT	−0.4	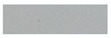	85.41	−5.95	3.44	0.311	0.341	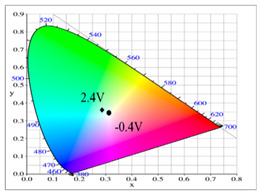
	0.8	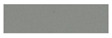	82.06	−8.31	8.14	0.316	0.353	
	1.0	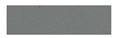	73.75	−12.33	10.88	0.315	0.365	
	1.4	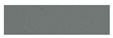	68.06	−14.97	7.90	0.304	0.363	
	2.4	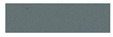	61.64	−18.83	3.63	0.285	0.357	
(d)								
P(bCmB-*co*-TF)/PEDOT	−0.3	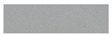	86.25	−6.88	4.39	0.311	0.343	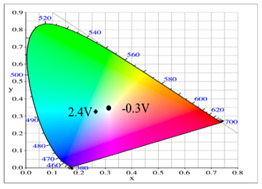
	1.2	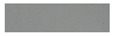	78.49	−9.49	7.62	0.313	0.354	
	1.6	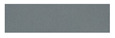	68.04	−14.27	3.33	0.295	0.349	
	1.9	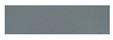	63.71	−15.82	−2.15	0.278	0.337	
	2.4	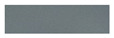	59.47	−16.45	−7.49	0.262	0.322	

**Table 6:** Electrochromic switching properties investigated at specific wavelengths for four devices.

ECDs	*λ* (nm)	*N*	*T*_ox_	*T*_red_	∆*T*	△OD	Q*_d_* (mC cm^−2^)	*η* (cm^2^∙C^−1^)	τ_c_ (s)	τ_b_ (s)
PbCmB/PEDOT	640	2	29.5	64.8	35.3	0.341	1.45	305.8	1.8	1.8
		60	34.1	67.2	33.1	0.295	1.33	288.3	1.8	1.9
P(bCmB-*co*-bTP)/PEDOT	635	2	18.0	58.7	40.7	0.514	1.56	428.4	1.9	1.8
		60	20.9	58.4	37.5	0.447	1.47	395.3	1.9	2.5
P(bCmB-*co*-dbBT)/PEDOT	635	2	25.7	62.1	36.4	0.383	1.46	341.0	1.8	1.9
		60	28.7	62.6	33.9	0.339	1.32	333.8	1.8	2.8
P(bCmB-*co*-TF)/PEDOT	640	2	26.8	62.5	35.7	0.368	1.51	316.8	1.9	1.9
		60	28.9	63.6	34.7	0.342	1.46	304.5	1.8	1.6
